# Delamanid Coadministered with Antiretroviral Drugs or Antituberculosis Drugs Shows No Clinically Relevant Drug-Drug Interactions in Healthy Subjects

**DOI:** 10.1128/AAC.00509-16

**Published:** 2016-09-23

**Authors:** Suresh Mallikaarjun, Charles Wells, Carolyn Petersen, Anne Paccaly, Susan E. Shoaf, Shiva Patil, Lawrence Geiter

**Affiliations:** Otsuka Pharmaceutical Development & Commercialization, Inc., Princeton, New Jersey, USA

## Abstract

Delamanid is a medicinal product approved for treatment of multidrug-resistant tuberculosis. Three studies were conducted to evaluate the potential drug-drug interactions between delamanid and antiretroviral drugs, including ritonavir, a strong inhibitor of CYP3A4, and selected anti-TB drugs, including rifampin, a strong inducer of cytochrome P450 (CYP) isozymes. Multiple-dose studies were conducted in parallel groups of healthy subjects. Plasma samples were analyzed for delamanid, delamanid metabolite, and coadministered drug concentrations, and pharmacokinetic (PK) parameters were determined. The magnitude of the interaction was assessed by the ratio of the geometric means and 90% confidence intervals. Coadministration of delamanid with tenofovir or efavirenz did not affect the PK characteristics of delamanid. Coadministration of Kaletra (lopinavir/ritonavir) with delamanid resulted in an approximately 25% higher delamanid area under the concentration-time curve from time 0 to the end of the dosing interval (AUCτ). Tenofovir, efavirenz, lopinavir, and ritonavir exposure were not affected by delamanid. Coadministration of delamanid with the TB drugs (ethambutol plus Rifater [rifampin, pyrazinamide, and isoniazid]) resulted in lower delamanid exposures (47 and 42% for the AUCτ and C_max_ [maximum concentration of a drug in plasma] values, respectively), as well as decreased exposure of three primary metabolites (approximately 30 to 50% lower AUCτ values). Delamanid did not affect rifampin, pyrazinamide, and isoniazid exposure; the ethambutol AUCτ and *C*_max_ values were about 25% higher with delamanid coadministration. The lack of clinically significant drug-drug interactions between delamanid and selected antiretroviral agents (including the strong CYP inhibitor ritonavir) and a combination of anti-TB drugs was demonstrated. Although there was a decrease in the delamanid concentrations when coadministered with ethambutol plus Rifater, this is likely related to decreased delamanid absorption and not to CYP induction.

## INTRODUCTION

Multidrug-resistant tuberculosis (MDR-TB), or tuberculosis resistant to two first-line drugs, isoniazid and rifampin, has emerged over the past 3 decades to greatly complicate efforts to control the disease. Current estimates are that approximately 480,000 cases of MDR-TB occur annually, or more than 5% of the nearly 9.6 million people estimated to develop TB each year (1, 2). MDR-TB is difficult to treat and usually requires four to six medications, including the more toxic and less potent second-line drugs administered for up to 2 years. In addition, treatment is further complicated among MDR-TB patients with HIV coinfection. These patients require additional treatment with antiretroviral medications to have a better chance at survival (3).

Delamanid is an anti-TB agent from the nitro-dihydro-imidazole class of compounds that inhibits mycolic acid synthesis in the Mycobacterium tuberculosis cell wall. In preclinical development, delamanid showed potent *in vitro* and *in vivo* activity against both drug-susceptible and drug-resistant strains of M. tuberculosis (4). In clinical development, delamanid showed measurable activity in early bactericidal trials in drug-susceptible TB patients (5). In MDR-TB patients, treatment with delamanid in combination with an optimized background regimen for 2 months significantly improved 2-month sputum culture conversion by approximately 50% compared to treatment with a placebo plus an optimized background regimen (6). In addition, in a longer-term observational study, delamanid plus an optimized background regimen treatment for ≥6 months was associated with higher favorable treatment outcomes compared to ≤2 months of treatment (74.5% versus 55%, *P* < 0.001) (7) and significantly lower mortality (12.0% versus 2.9%, *P* = 0.001) (8). Based on these results, delamanid was approved in the European Union, Japan, and the Republic of Korea in 2014 for the treatment of pulmonary MDR-TB in adult patients. The recommend dose of delamanid is 100 mg bid to be taken with food.

In the combined treatment of TB patients and MDR-TB patients coinfected with HIV, the risk of clinically significant drug-drug interactions increases, especially when considering the number of commonly coadministered anti-TB and antiretroviral medications that are either inducers or inhibitors of CYP, including newer anti-TB drugs such as bedaquiline and PA-824 (9, 10). Among the commonly coadministered drugs, rifampin (11) is a strong inducer of CYP450 isozymes, efavirenz (12) has been shown to be a moderate inducer of CYP3A4, and ritonavir (13) is a strong inhibitor of CYP3A. The metabolism of isoniazid is mediated by *N*-acetyltransferase, which undergoes genetic polymorphism, leading to extensive and poor metabolizer phenotypes (14). Delamanid is primarily metabolized by albumin to DM-6705; the metabolism of DM-6705 to other metabolites is thought to involve pathways mediated by CYP (15). Although there are drugs that are partially metabolized by albumin, the metabolism as a primary pathway of drugs by albumin is unique. Therefore, although clinically relevant drug-drug interactions with inhibitors and inducers of CYP isoenzymes were not expected with delamanid, interactions due to some CYP involvement could not be ruled out.

We report here the results using two broad categories of medications evaluated for potential drug-drug interactions in the delamanid development program: first-line anti-TB drugs (including the strong CYP3A4 and other CYP450 isoenzymes inducer rifampin) and antiretroviral drugs used in HIV-infected patients (including the moderate CYP3A4 inducer efavirenz) and the strong CYP3A4 inhibitor ritonavir.

## MATERIALS AND METHODS

### Study design.

All studies were performed in accordance with Title 21 of the US Code of Federal Regulations Part 50 and Part 56 and in compliance with the International Conference on Harmonization-Good Clinical Practice (16), the sponsor's standard operating procedures, and ethical principles for the protection of human research subjects that have their origins in the Declaration of Helsinki. The study protocols, amendments, and informed consent forms were reviewed and approved by the governing institutional review board of each investigational center prior to starting the study. Written informed consent was obtained from all subjects before any study-related procedures were performed.

### (i) Study 1.

Study 1 was a phase 1, randomized, double-blind, placebo-controlled, drug-drug interaction study following multiple once daily oral doses in three parallel groups of clinic-confined healthy subjects receiving either (i) delamanid, (ii) ethambutol plus Rifater (ethambutol-Rifater), or (iii) delamanid plus ethambutol-Rifater. Rifater is a combination tablet of rifampin, isoniazid, and pyrazinamide. The study was conducted at PPD Development, LP, in Austin, TX.

### (ii) Study 2.

Study 2 was a phase 1, randomized, open-label, oral multiple-dose drug interaction study in seven parallel groups of clinic-confined healthy subjects. Delamanid (twice-daily dosing), tenofovir, efavirenz, or Kaletra (lopinavir/ritonavir) were administered alone, and delamanid was also coadministered with tenofovir, efavirenz, or Kaletra for 14 days. The study was conducted at PPD Development, LP, in Austin, TX. The efavirenz arms (alone and with delamanid) were discontinued midstudy due to adverse events (AEs) and a revised design tested in study 3.

### (iii) Study 3.

Study 3 was a phase 1, randomized, open-label, modified sequential, oral multiple-dose drug interaction study in two parallel groups of clinic-confined healthy subjects. Subjects were administered either efavirenz for 10 days (group 1) or delamanid twice daily for 7 days, followed by delamanid twice daily plus efavirenz for 10 days (group 2). The study was conducted at Covance Clinical Research Unit in Evansville, IN.

### Subjects.

All subjects enrolled in each study underwent a medical evaluation prior to the initiation of study treatment. These assessments included a review of relevant medical history and concomitant medications, physical examination, vital signs, body weight and height, 12-lead electrocardiogram (ECGs), and clinical laboratory tests. The subjects were evaluated to assure they met the inclusion and exclusion criteria listed below.

### (i) Study 1.

Healthy male or female subjects between 18 and 45 years of age, who weighed ≥55 kg and had a body mass index (BMI) between 19 and 32 kg/m^2^, were surgically sterile or willing to remain abstinent or to practice double-barrier forms of birth control and were able to provide written informed consent. Subjects were in good health, as determined by medical history, physical examination, ECG, serum/urine biochemistry, hematology, and serology tests. The main exclusion criteria were (i) clinically significant abnormalities in blood pressure, heart rate, and ECG reading (including QTc > 450 ms), (ii) a history of any significant drug allergy or a known or suspected drug hypersensitivity to any of the drugs being studied, (iii) use of tobacco products or having had daily exposure to second-hand smoke within 2 months prior to the screening visit, (iv) use of any prescription, over-the-counter, or herbal medication, or vitamin supplements within 14 days prior to dosing, or antibiotics within 30 days prior to dosing, (v) history of a positive urine alcohol test and/or urine drug screen for substances of abuse at screening or upon admission to the study center, (vi) consumption of alcohol and/or food and beverages containing methylxanthines, grapefruit, grapefruit juice, Seville oranges, or Seville orange juice within 72 h prior to dosing, (vii) being unable to consume the standard meal, (viii) having taken an investigational drug or donated blood or plasma within 30 days of dosing, (ix) a history of or current hepatitis or AIDS or being a carrier of HBsAg and/or anti-HCV or HIV antibodies, or (x) a history of prior exposure to delamanid.

### (ii) Study 2.

Study 2 was similar to study 1, except there was no minimum weight requirement, subjects were to have a BMI ≥ 15 and ≤ 32 kg/m^2^, subjects had to be HIV negative, and subjects were excluded if either their QTcF or QTcB interval was over 430 ms in male subjects or over 450 ms in female subjects. In addition, previous illicit drug consumption, a medical history of psychiatric illness, or a diagnosis or significant symptoms of psychiatric illness were added as exclusion criteria, and a mental/neuropsychiatric status examination was also part of the scheduled assessments after the efavirenz treatment arms were discontinued.

### (iii) Study 3.

Study 3 was similar to study 1, except there was no minimum weight requirement, subjects were to have a BMI of ≥18 and ≤32 kg/m^2^, subjects were to be HIV negative, and subjects were excluded if their QTcF interval was over 450 ms in male subjects or over 470 ms in female subjects. As in study 2, previous illicit drug consumption, a medical history of psychiatric illness, or a diagnosis or significant symptoms of psychiatric illness were exclusion criteria, and a mental/neuropsychiatric status examination was also part of the scheduled assessments.

### Treatment and dosing regimens. (i) Study 1.

Delamanid and placebo tablets were manufactured by Otsuka Pharmaceutical Co., Ltd. (Japan). Ethambutol was supplied as commercially packaged Myambutol tablets (X-Gen Pharmaceuticals, Big Flats, NY) in strengths of 100 and 400 mg. Rifater was provided as commercially packaged tablets (Sanofi-Aventis, Bridgewater, NJ) that contained 120 mg of rifampin, 50 mg of isoniazid, and 300 mg of pyrazinamide per tablet. Pyridoxine was supplied as a commercially packaged generic drug product with 25 mg of pyridoxine per tablet (VersaPharm, Inc., Marietta, GA).

Subjects were treated once daily for 15 days (days 1 through 15) with delamanid and ethambutol-Rifater, with delamanid and placebo for ethambutol-Rifater, or with placebo for delamanid and ethambutol-Rifater. The administration of ethambutol-Rifater was continued for an additional 4 days (days 16 through 19). The dose of delamanid was 200 mg (four 50-mg tablets), the dose of ethambutol was 1100 mg (two 400-mg tablets and three 100-mg tablets), and the dose of Rifater was 720 mg of rifampin, 300 mg of isoniazid, and 1,800 mg of pyrazinamide (six tablets). In addition, subjects received 25 mg of pyridoxine once daily on days 1 through 19 as prophylaxis against the development of peripheral neuropathy from isoniazid administration. Labeling for ethambutol (17) and Rifater (18) indicates that food decreases bioavailability. Therefore, ethambutol and Rifater (or placebo) were administered on an empty stomach, 1 h before a standard breakfast. One hour after administration of ethambutol-Rifater, subjects consumed a standard breakfast within 20 min. Dosing with delamanid (19) (or placebo) followed the meal and was administered within 30 min of beginning the standard breakfast. All doses were administered from a cup with a closed lid to prevent subjects from inspecting the tablets and were given orally with 240 ml of room temperature still water.

### (ii) Study 2.

Delamanid tablets were manufactured by Otsuka Pharmaceutical Co., Ltd. (Japan). Tenofovir was supplied as commercially available Viread manufactured by Gilead Sciences, Inc. (Foster City, CA), and lopinavir/ritonavir was supplied as commercially available Kaletra manufactured by Abbott Laboratories (Abbott Park, IL). Subjects were randomly assigned to receive one of the five following treatments for 14 days: 100 mg of delamanid (two 50-mg tablets) twice daily, 300 mg of tenofovir (one 300-mg Viread tablet) once daily, 300 mg of tenofovir once daily plus 100 mg of delamanid twice daily, 400 mg of lopinavir and 100 mg of ritonavir (two 200/50-mg Kaletra tablets) twice daily, or 400 mg of lopinavir and 100 mg of ritonavir twice daily plus 100 mg of delamanid twice daily. All doses were given orally with 240 ml of room temperature still water. The once-daily dose or the morning dose of the twice-daily regimens was given within 30 min of the start of a standard meal. The evening dose of twice-daily regimens was given 12 h after the morning dose and within 30 min after the start of a standard meal.

### (iii) Study 3.

Delamanid tablets were manufactured by Otsuka Pharmaceutical Co., Ltd. (Japan). Efavirenz was supplied as commercially available Sustiva manufactured by Bristol-Myers Squibb (USA). Subjects were randomly assigned to receive one of the following two treatments: group 1 received 600 mg of efavirenz (one 600-mg Sustiva tablet) once daily for 10 days and group 2 received 100 mg of delamanid (two 50-mg tablets) twice daily for 7 days, followed by 100 mg of delamanid twice daily plus 600 mg of efavirenz once daily for 10 days, for a total of 18 days of dosing. Consistent with labeling, the once-daily dose of efavirenz in both groups was administered in the evening at about 8 p.m. on an empty stomach (2 h after a standard meal and 2 h prior to a snack) (20). The first dose of delamanid was the evening dose administered on day 1, and the last delamanid dose was the evening dose administered on day 18. Each evening dose of delamanid was administered at about 6 p.m., and each morning dose of delamanid was administered at about 8 a.m.; all delamanid doses were given within 30 min of the start of a standard meal.

### Safety assessments.

Safety assessments performed in these studies were consistent with well-monitored phase 1 healthy subject studies and included physical examination, vital signs, 12-lead ECG readings, clinical laboratory tests, and the collection of adverse events. Safety assessments were also carried out at the end of the study treatment and follow-up period (or early withdrawal for any reason). Additional safety assessments were done whenever deemed appropriate by the investigator. In study 1, visual acuity assessments were made on days 0, 7, and 15 (consistent with labeling for ethambutol) (17), and ECG examinations were performed over a 24-h period at the same time each day on days 0 and 15 to allow for time-matched change from baseline analysis of potential QT prolongation effects.

### Pharmacokinetic sampling. (i) Study 1.

Blood draws for the determination of plasma delamanid and delamanid metabolite concentrations were obtained predose on days 1, 10, 11, 12, 13, 14, and 15 and postdose at 1, 2, 3, 4, 5, 6, 8, 12, 24, 48, 72, 96, and 120 h after the last dose of delamanid or placebo on day 15. Blood draws for the determination of plasma ethambutol, rifampin, pyrazinamide, and isoniazid concentrations were obtained prior to the first dose of ethambutol-Rifater or placebo on day 1, and 1.5 h postdose on days 10, 11, 12, 13, and 14, and on day 15 at the following times: predose and then at 0.25, 0.5, 1, 1.5, 2.5, 3.5, 4.5, 5.5, 6.5, 7.5, 9.5, 13.5, and 24 h postdose.

### (ii) Study 2.

Blood draws for the determination of plasma delamanid and delamanid metabolites, tenofovir, ritonavir, and lopinavir concentrations were obtained predose on days 1, 12, and 13. On day 14, blood samples were obtained predose and at 1, 2, 3, 4, 5, 6, 8, 12, 16, and 24 h after the morning dose.

### (iii) Study 3.

For group 1, blood draws for the determination of plasma efavirenz concentrations were obtained on predose days 1, 8, 9, and 10, and at 1, 2, 3, 4, 5, 6, 12, 16, and 24 h after the day 10 dose. For group 2, blood draws for the determination of plasma delamanid and delamanid metabolite concentrations were obtained prior to the delamanid evening dose on days 1, 5, 6, and 7 and at 1, 2, 3, 4, 5, 6, 14, 18, and 24 h after the day 7 delamanid evening dose. On day 17, samples were obtained prior to the evening dose and at 1, 2, 3, 4, 5, 6, 14, 18, and 24 h after the day 17 evening dose. Blood draws for the determination of plasma efavirenz concentrations were obtained prior to the delamanid evening dose on days 1 and 8 and prior to the efavirenz dose on days 15, 16, and 17. On day 17, samples were also obtained at 1, 2, 3, 4, 5, 6, 12, 16, and 24 h after the efavirenz dose.

### Bioanalytical methods.

Validated methods and the performance of the assays are summarized below. Each validated method had adequate linearity, sensitivity, precision, and accuracy to meet the objectives of these three drug-drug interaction studies. No interference was observed when delamanid and its metabolites were quantitated in the presence of rifampin, 25-desacetylrifampicin, ethambutol, and isoniazid, as well as when these analytes were quantitated in the presence of delamanid and all its metabolites.

### (i) Study 1.

Plasma samples were analyzed for delamanid and its metabolites using a specific and validated high performance liquid chromatography assay with a tandem mass spectrophotometric detection (i.e., liquid chromatography-tandem mass spectrometry [LC-MS/MS]) method developed by Tandem Laboratories, Salt Lake City, UT (21). Delamanid, its metabolites, and the internal standard (OPC-14714) were extracted from plasma using protein precipitation, followed by injection of the supernatant. The method was linear over the range between 1.00 and 500 ng/ml for delamanid and the metabolites, with calibration curve coefficients of determination (*r*^2^) of ≥0.9868 for all analytes. For each batch of samples processed, the calculated concentrations of at least two-thirds of the quality control (QC) samples were within 15% of nominal. At each QC concentration, the percent coefficient of variation (%CV) values were within 8.6%, and the percent bias values were within 5.0%.

Plasma samples were analyzed for ethambutol concentration using a specific and validated LC-MS/MS method by PRA International, The Netherlands. Ethambutol and the deuterated internal standard (d4-ethambutol) were extracted using protein precipitation, followed by injection of the supernatant. The method was linear over am ethambutol concentration range between 50.0 and 10,000 ng/ml, with calibration curve regression coefficients (*r*) of ≥0.9989. For each batch of samples processed, the calculated concentrations of at least two-thirds of the QC samples were within 15% of nominal. At each QC concentration, the %CV values were within 9.4%, and the percent bias values were within 1.9%.

Plasma samples were analyzed for rifampin and its metabolite 25-desacetyl rifampin concentration using a specific and validated high-pressure liquid chromatography (HPLC) with UV detection method by Tandem Laboratories. Rifampin, its metabolite, and the internal standard (sulindac) were extracted from plasma using solid-phase extraction. The method was linear over a range between 0.200 and 40.0 μg/ml for rifampin and between 0.100 and 5.00 μg/ml for the metabolite, with calibration curve *r* ≥ 0.9998. For each batch of samples processed, the calculated concentrations of at least two-thirds of the QC samples were within 15% of nominal. At each QC concentration, the %CV values were within 3.8%, and the percent bias values were within 6.3% for both analytes.

Plasma samples were analyzed for isoniazid and pyrazinamide concentration using a specific and validated HPLC/UV method developed by PRA International. Isoniazid, pyrazinamide, and the internal standard (nicotinamide) were extracted from plasma using liquid-liquid extraction. The method was linear over a range between 0.0500 and 15.0 μg/ml for isoniazid and between 0.500 and 100 μg/ml for pyrazinamide, with calibration curve *r* ≥ 0.9995 for both analytes. For each batch of samples processed, the calculated concentrations of at least two-thirds of the QC samples were within 15% of nominal. At each QC concentration, the %CV values were within 3.6% for isoniazid and pyrazinamide, and the percent bias values were within 2.3% for both analytes. The *N*-acetyltransferase (NAT2) genotype to support isoniazid metabolism interpretation and the CYP2C9 genotype to assess delamanid metabolism interpretation were determined by Gentris Clinical Genetics, Inc. (Morrisville, NC).

### (ii) Study 2.

Plasma samples were analyzed for delamanid and metabolite concentration using a specific and validated UPLC-MS/MS method by Tandem Laboratories similar to that described for study 1 (21). Plasma samples were analyzed for lopinavir and ritonavir concentration using a specific and validated LC-MS/MS method by Tandem Laboratories. Lopinavir, ritonavir, and their deuterated internal standards were extracted from plasma using solid-phase extraction, followed by injection of the supernatant. The samples were quantitated against calibration standards prepared in plasma and processed like the samples. The method was linear over the range between 5.00 and 5,000 ng/ml for lopinavir and ritonavir, with a calibration curve of *r*^2^ ≥ 0.9868 for both analytes. For each batch of samples processed, the calculated concentrations of at least two-thirds of the QC samples were within 15% of nominal. At each QC concentration, the %CV values were within 4.5%, and the percent bias values were within 4.7% for both analytes.

Plasma samples were analyzed for tenofovir concentration using a specific and validated LC-MS/MS method developed by Tandem Laboratories. The samples were quantitated against calibration standards prepared in plasma and processed like the samples. The method was linear over the range between 1.00 and 500 ng/ml tenofovir, with calibration curve of *r*^2^ ≥ 0.9868. For each batch of samples processed, the calculated concentrations of at least two-thirds of the QC samples were within 15% of nominal. At each QC concentration, %CV values were within 14.3%, and the percent bias values were within 10.3%.

### (iii) Study 3.

Plasma samples were analyzed for delamanid and its metabolites concentration by Tandem Laboratories using the UPLC-MS/MS method described for study 2, but only quantifying the three primary metabolites: DM-6704, DM-6705, and DM-6706. Plasma samples were analyzed for efavirenz concentration using a specific and validated LC-MS/MS method (Tandem Laboratories). The method was linear over the range between 10.0 and 2000 ng/ml for efavirenz, with a calibration curve of *r*^2^ ≥ 0.9979. For each batch of samples processed, the calculated concentrations of at least two-thirds of the QC samples were within 15% of nominal. At each QC concentration, the %CV values were within 5.8%, and the percent bias values were within 2.0%. The CYP2B6* genotype was determined using the Affymetrix DMET Plus Array by Covance Genomics Laboratory LLC, Seattle, WA.

### Pharmacokinetic and statistical analysis.

In all three studies, the concentration values below the quantitation limit were set to zero for the PK parameter calculations and descriptive statistics. Actual blood sample times were used for PK calculations, and the PK plasma concentration-time data were analyzed using a noncompartmental method (22). Values for *C*_max_ and *T*_max_ were determined directly from the observed data. The AUC values were determined using the linear trapezoidal rule. AUC from time 0 to the end of the dosing interval (AUCτ) was estimated from data after the last dose in these multiple-dose studies.

PK calculations were performed with WinNonlin version 4.0 for study 1 and version 5.2 for studies 2 and 3 (Pharsight Corporation, Princeton, NJ). Descriptive statistics for plasma concentrations by treatment and time point and PK parameters by treatment were determined by using S-Plus version 6.1 for studies 1 and 2 (Insightful Corporation, Palo Alto, CA) and by SAS version 9.1.3 for study 3 (SAS, Cary, NC). For all studies, the primary PK analysis variables were *C*_max_ and AUCτ for delamanid and coadministered drugs. Statistical analyses were performed using the log-transformed data of *C*_max_ and AUCτ. The magnitude of the drug-drug interaction was assessed by computing the ratio of the geometric means (GMR) of the drug in combination versus alone and the corresponding 90% confidence intervals (CIs) based on the log-transformed data. In study 1, for delamanid, the ratio of delamanid and ethambutol plus the fixed-dose combination of Rifater to delamanid alone was determined. For ethambutol, the ratio of ethambutol-Rifater plus delamanid to ethambutol-Rifater alone was determined. Similar analyses were performed for each additional drug in the regimen (rifampin, isoniazid, and pyrazinamide). For studies 2 and 3, the magnitude of the drug-drug interaction was assessed by computing the GMR and the corresponding 90% CIs based on the log-transformed data similar to study 1.

The interpretation of the confidence intervals was based on the article by Williams et al. (23), where four possible outcomes are possible: (i) equivalence is documented (the 90% CIs are within the 0.8 to 1.25 limits), (ii) equivalence is suggested (where one of the intervals is outside the boundary, but the GMR is within the boundary), (iii) nonequivalence is documented (the 90% CIs are outside the boundaries), and (iv) nonequivalence is suggested (where both the GMR and one of the intervals are outside the boundary).

## RESULTS

### Subject population.

Demographic characteristics of the healthy subjects enrolled in the three drug-drug interaction studies are shown in Table 1 by study and treatment group. Subjects were mostly white (about 70% of the subjects enrolled), non-Hispanic (50% to 93%), and male (50% to 67%), and they ranged in age from 18 to 45 years. Overall, the healthy subject populations were similar in the three studies.

**TABLE 1:** Demographic data summary for healthy subjects enrolled in three drug-drug interaction studies^*a*^

Demographic characteristic	Statistic	Study 1 treatment			Study 2 treatment					Study 3 treatment	
		DLM + PLC	DLM + EMB + Rifater	PLC + EMB + Rifater	DLM	TDF	DLM + TDF	KAL	DLM + KAL	DLM (+ EFV)	EFV
No. of subjects	*N*	14	22	19	15	17	18	14	16	15	15
Age (yr)	Mean ± SD	30.6 ± 8.2	30.5 ± 7.9	29.7 ± 6.0	28.0 ± 6.0	29.0 ± 7.0	35.0 ± 6.0	27.0 ± 6.0	30.0 ± 7.0	30.3 ± 6.9	28.1 ± 6.6
	Range	18–44	18–45	22–41	18–37	21–41	25–45	18–35	21–44	18–42	20–44
Wt (kg)	Mean ± SD	73.9 ± 13.1	78.9 ± 13.7	77.8 ± 13.5	75.0 ± 17.0	72.0 ± 9.0	74.0 ± 10.0	71.0 ± 11.0	76.0 ± 13.0	78.0 ± 17.7	74.4 ± 11.9
	Range	57.0–93.9	55.6–101	55.3–103	55–104	54–89	51–92	57–92	56–105	53.6–106	55.2–96.3
Ht (cm)	Mean ± SD	169.7 ± 10.4	168 ± 10.9	172.2 ± 11.4	170.0 ± 10.0	168.0 ± 8.0	167.0 ± 10.0	169.0 ± 8.0	169.0 ± 11.0	173.2 ± 9.7	170.6 ± 8.1
	Range	157–191	150–187	145–189	158–186	156–182	151–184	151–181	156–188	156–186	157–184
Gender	*n* (%)										
Male		9 (64.3)	13 (59.1)	12 (63.2)	8 (53.3)	9 (52.9)	9 (50.0)	8 (57.1)	9 (56.3)	10 (66.7)	9 (60.0)
Female		5 (35.7)	9 (40.9)	7 (36.8)	7 (46.7)	8 (47.1)	9 (50.0)	6 (42.9)	7 (43.7)	5 (33.3)	6 (40.0)
Race	*n* (%)										
White		10 (71.4)	16 (72.7)	13 (68.4)	11 (73.3)	13 (76.5)	13 (72.2)	10 (71.4)	12 (75.0)	7 (46.7)	12 (80.0)
Black		3 (21.4)	6 (27.3)	6 (31.6)	3 (20.0)	3 (17.6)	5 (27.8)	4 (28.6)	2 (12.5)	5 (33.3)	1 (6.7)
Asian		1 (7.1)	0	0	1 (6.7)	1 (5.9)	0	0	1 (6.3)	1 (6.7)	1 (6.7)
Other		0	0	0	0	0	0	0	1 (6.3)	2 (13.3)	1 (6.7)
Ethnicity	*n* (%)										
Hispanic/Latino		4 (30.8)	7 (31.8)	4 (21.1)	5 (33.3)	5 (29.4)	9 (50.0)	5 (35.7)	7 (43.8)	2 (13.3)	1 (6.7)
Non-Hispanic/Latino		9 (69.2)	15 (68.2)	15 (78.9)	10 (66.7)	12 (70.6)	9 (50.0)	9 (64.3)	9 (56.3)	13 (86.7)	14 (93.3)

a DLM, delamanid; EFV, efavirenz; EMB, ethambutol; KAL, Kaletra (a combination tablet of ritonavir and lopinavir); *n*, number of subjects in the subset; *N*, total number of subjects enrolled in a treatment group; PLC, placebo; Rifater, a combination tablet of rifampin, pyrazinamide, and isoniazid; TDF, tenofovir; %, *n*/*N*. The study 2 data do not include either of the efavirenz treatment groups that were stopped early in the trial and so did not yield pharmacokinetic data.

### Subject disposition. (i) Study 1.

A total of 55 subjects were randomized and received at least one dose of study drug and thus were analyzed for safety: 14 in the delamanid plus placebo group, 22 in the delamanid and ethambutol-Rifater group, and 19 in the placebo and ethambutol-Rifater group. Overall, 25/55 subjects (45.5%) discontinued the study, 21/55 subjects (38.2%) due to adverse events (AEs) and 4/55 (7.3%) due to investigator withdrawal of the subject. Most of the subjects (19/21) who discontinued due to an AE experienced a generalized rash after receiving the first dose of ethambutol-Rifater and were discontinued prior to receiving any delamanid or placebo for delamanid. The greater than planned number of subjects, as well as the imbalance in the number randomized per group, reflects the decision to replace 16 subjects who either withdrew due to a generalized rash (known to be a tolerance issue with Rifater) (18) or who were withdrawn by the investigator because of a suspected decrease in visual acuity, which was shown on further investigation to be a false-positive event. No AEs related to visual acuity changes were reported. No clinically relevant changes in safety ECGs were noted over time for subjects in any treatment group. The maximum time-matched mean change from baseline in individually corrected QT intervals (QTcI) was lower for the delamanid and ethambutol-Rifater group compared to the delamanid plus placebo group (3.4 ms versus 8.5 ms). Excluding the high incidence of generalized rash in subjects treated with a single dose of ethambutol-Rifater observed in this study, overall, multiple once-daily oral doses of 200 mg of delamanid alone and in combination with ethambutol and rifampin/isoniazid/pyrazinamide were well tolerated.

### (ii) Study 2.

A total of 89 subjects were randomized and received at least one dose of study drug and thus were analyzed for safety: 15 in the delamanid-only group, 5 in the efavirenz-only group, 4 in the efavirenz plus delamanid group, 17 in the tenofovir-only group, 18 in the tenofovir plus delamanid group, 14 in the lopinavir/ritonavir-only group, and 16 in the lopinavir/ritonavir plus delamanid group. All of these subjects were included in the safety analysis. The original protocol had a treatment group receiving efavirenz (dosed in the morning) and another group receiving coadministered efavirenz (dosed in the morning) and delamanid (dosed twice daily). Nine subjects were in these groups. Subjects receiving efavirenz alone or delamanid plus efavirenz experienced central nervous system (CNS)-related AEs. These CNS events were likely due to administering efavirenz in the morning and resulted in the cessation of dosing in these two treatment arms, early discontinuation of subjects in the other treatment arms in the study, and an amended protocol with five treatments (reported here as study 2). The incidence of AEs among subjects taking delamanid coadministered with the antiretroviral drugs tenofovir and lopinavir/ritonavir was comparable to that in patients taking the antiretroviral drugs alone. Overall, the combinations of delamanid with either tenofovir or with lopinavir and ritonavir were well tolerated.

### (iii) Study 3.

As a result of the neuropsychiatric AEs observed with the use of efavirenz plus delamanid in study 2, the two efavirenz arms were suspended, and a new study (Study 3) was designed with enhanced safety features (including dosing efavirenz in the evening on an empty stomach, consistent with labeling, and exclusion of patients with a prior psychiatric or drug abuse problem) to further explore the potential for drug-drug interaction. A total of 30 subjects were randomized and received at least one dose of study drug and thus were analyzed for safety: 15 in the efavirenz-only group and 15 in the group receiving delamanid alone for 7 days, followed by delamanid plus efavirenz for 10 days. Of the 30 subjects (86.7%), 26 completed the study. The administration of efavirenz alone, delamanid alone, and delamanid plus efavirenz were well tolerated, although the overall incidence of AEs was higher during concomitant dosing compared to either medication alone. While a higher rate of neuropsychiatric AEs (e.g., euphoric mood and abnormal dreams) was observed with delamanid plus efavirenz compared to either drug alone, no subject discontinued the study because of neuropsychiatric events or had serious neuropsychiatric AEs.

### Pharmacokinetic results.

**FIG 1:**
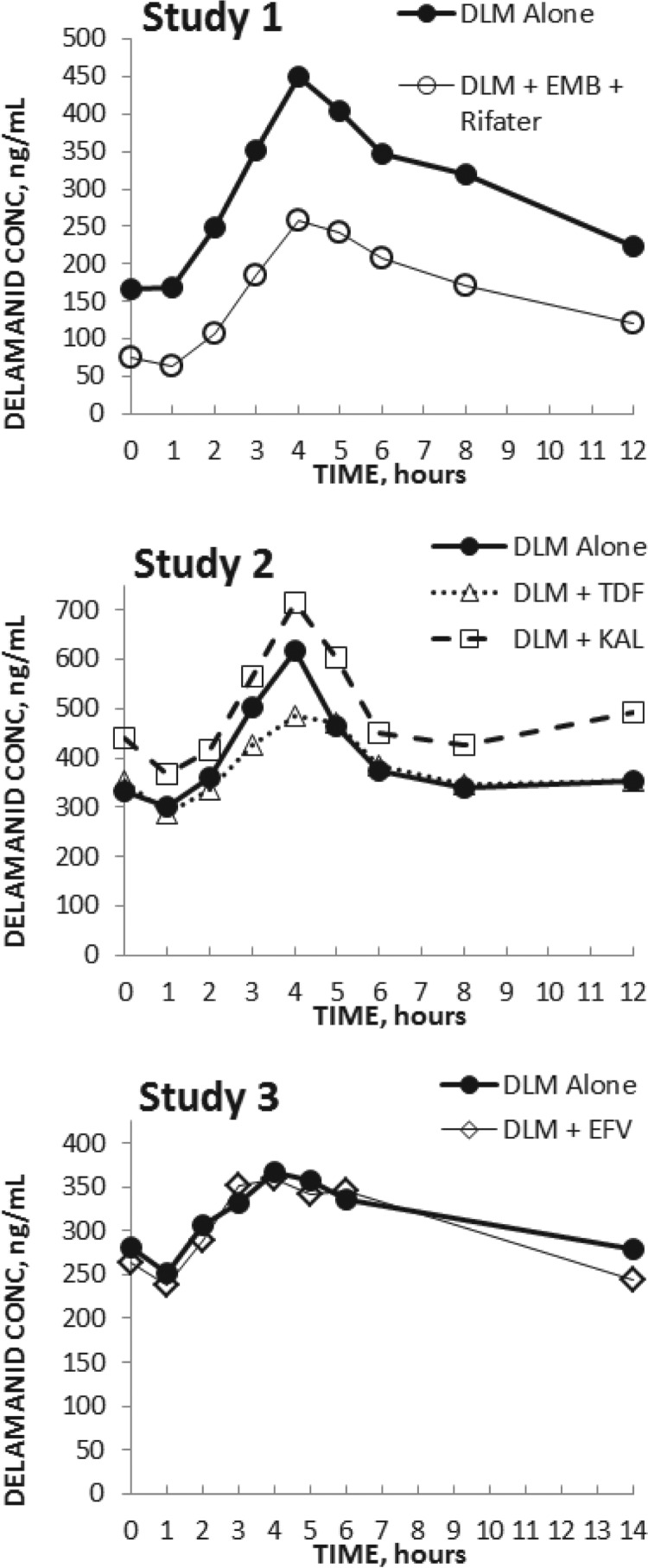
Delamanid plasma concentration-time profiles alone or with coadministered drugs. DLM, delamanid; EFV, efavirenz; EMB, ethambutol; KAL, Kaletra (lopinavir/ritonavir); TDF, tenofovir.

The delamanid plasma concentration-versus-time profiles after multiple dosing for delamanid alone or with each coadministered drug (by study) are shown in Fig. 1. Key PK parameters for delamanid, the number of PK evaluable subjects, and statistical evaluations of potential drug-drug interactions (by study) are presented in Table 2. PK parameters of coadministered drugs and statistical evaluation of potential drug-drug interactions (by study) are presented in Table 3.

**TABLE 2:** Delamanid pharmacokinetic parameters by treatment group and drug-drug interaction comparison^*a*^

Study	Treatment	*n*	Delamanid parameter estimate			GMR (90% CI)	
			*T*_max_ (h)	*C*_max_ (ng/ml)	AUCτ (ng·h/ml)	*C*_max_	AUCτ
1	DLM + PLC	13	4.00 (4.00–8.00)	476 ± 119	5,950 ± 1,440		
	DLM + EMB + Rifater	8	4.06 (4.00–5.00)	270 ± 38.9	3,110 ± 706	0.577 (0.492–0.676)	0.525 (0.439–0.628)
2	DLM alone	11	4.00 (4.00–4.00)	617 ± 135	4,700 ± 733		
	DLM + TDF	13	4.00 (3.00–8.05)	518 ± 114	4,510 ± 770	0.840 (0.714–0.989)	0.958 (0.835–1.099)
	DLM + KAL	12	4.00 (3.00–5.00)	734 ± 196	5,830 ± 1,660	1.177 (0.997–1.390)	1.216 (1.057–1.399)
3	DLM alone	14	4.00 (2.00–6.00)	391 (66.2)	4,382 ± 754		
	DLM + EFV	12	4.00 (0.00–6.02)	394 (72.9)	4,239 ± 775	0.995 (0.926–1.069)	0.968 (0.910–1.030)

a DLM, delamanid; EFV, efavirenz; EMB, ethambutol; KAL, Kaletra (a combination tablet of ritonavir and lopinavir); *n*, total number of subjects enrolled in a treatment group; NA, not applicable; PLC, placebo; Rifater, a combination tablet of rifampin, pyrazinamide, and isoniazid; TDF, tenofovir. The *C*_max_ and AUCτ data are presented as means ± the standard deviations; the *T*_max_ data are presented as medians, with the minimum to maximum range in parentheses. GMR (90% CI) = delamanid plus concomitant drug to delamanid alone.

**TABLE 3:** Pharmacokinetic parameters of coadministered drugs and drug-drug interaction comparison^*a*^

Coadministered drug (study no.)	*C*_max_ (μg/ml)			AUCτ (h·µg/ml)		
	Without DLM (*n*)	With DLM (*n*)	GMR (90% CI)	Without DLM (*n*)	WITH DLM (*n*)	GMR (90% CI)
Isoniazid (1)	5.54 ± 1.69 (9)	4.62 ± 0.98 (8)	0.854 76–1.0 (0.679)	20.0 ± 8.0 (9)	12.1 ± 6.9 (8)	0.588^*b*^ (0.399–0.867)
Rifampin (1)	11.2 ± 3.87 (9)	13.2 ± 5.41 (8)	1.141 (0.775–1.677)	48.2 ± 18.3 (9)	55.9 ± 28.6 (8)	1.071 (0.687–1.670)
Pyrazinamide (1)	49.7 ± 10.4 (9)	51.4 ± 9.06 (8)	1.043 (0.876–1.243)	488 ± 90.3 (9)	533 ± 141 (8)	1.074 (0.886–1.303)
Ethambutol (1)	3.56 ± 0.93 (9)	4.45 ± 0.82 (8)	1.268 (1.046–1.538)	18.2 ± 3.21 (9)	22.4 ± 4.77 (8)	1.226 (1.043–1.441)
Tenofovir (2)	0.326 ± 0.069 (12)	0.294 ± 0.076 (13)	0.894 (0.768–1.040)	3.130 ± 0.730 (12)	2.850 ± 0.644 (13)	0.914 (0.781–1.068)
Lopinavir (2)	12.9 ± 3.17 (11)	13.6 ± 3.38 (12)	1.050 (0.880–1.254)	112 ± 22.5 (11)	118 ± 33.0 (12)	1.036 (0.864–1.244)
Ritonavir (2)	1.30 ± 0.68 (11)	1.27 ± 0.80 (12)	0.959 (0.657–1.399)	5.83 ± 1.94 (11)	6.20 ± 2. 94 (12)	1.031 (0.773–1.373)
Efavirenz (3)	5.95 ± 1.67 (14)	5.81 ± 3.03 (12)	0.937 (0.754–1.165)	84.7 ± 37.8 (14)	83.1 ± 57.8 (12)	0.937 (0.715–1.228)

a GMR (90% CI) = concomitant drug plus delamanid versus concomitant drug alone. DLM, delamanid. *C*_max_ and AUCτ data are presented as means ± the standard deviations. 90% CI, 90% confidence interval; *n*, number of subjects.

b The GMR data presented for isoniazid are confounded by the NAT2 genotype since the groups were not matched for slow and rapid acetylators (unpublished data).

### (i) Study 1.

Delamanid concentrations reached steady state by day 15, the last day of dosing, following 200-mg once-daily dosing of delamanid alone or with ethambutol-Rifater. As expected, given the long half-life of metabolites (19), delamanid metabolite concentrations did not yet reach steady state with the 15-day duration of the study. Based on the criteria of Williams et al., nonequivalence of steady-state delamanid *C*_max_ and AUCτ was documented when coadministered with ethambutol-Rifater (*C*_max_ geometric mean ratio [GMR] = 0.577 [90% CI = 0.492 to 0.676] and AUCτ GMR = 0.525 [90% CI = 0.439 to 0.628]) (Table 2). The concentrations of the primary and most prevalent metabolites of delamanid in this study (DM-6704, DM-6705, and DM-6706) were also about 30 to 50% lower (based on the AUC) when delamanid was coadministered with ethambutol-Rifater (Table 4). The mean day 15 AUC ratio of metabolite DM-6704 to delamanid and the ratio of metabolite DM-6705 to delamanid were similar between treatments. This observation, coupled with the overall lower concentrations of metabolites, suggests that induction of CYP3A4 by rifampin does not play a major role in the observed lower delamanid exposure with combination treatment and that reduced bioavailability of delamanid may occur when delamanid is coadministered with ethambutol-Rifater under the conditions of this study. CYP2C9 genotype had no effect on delamanid PK (unpublished results).

**TABLE 4:** Delamanid metabolite pharmacokinetic parameters with or without ethambutol-Rifater in study 1^*a*^

Treatment metabolite	Mean ± SD			
	DLM + PLC (*n* = 13)		DLM + EMB + Rifater (*n* = 8)	
	*C*_max_ (ng/ml)	AUCτ (ng·h/ml)	*C*_max_ (ng/ml)	AUCτ (ng·h/ml)
DM-6704	63.5 ± 23.4	1,290 ± 477	32.7 ± 14.2	667 ± 262
DM-6705	55.3 ± 13.6	1,140 ± 311	40.7 ± 13.3	799 ± 227
DM-6706	45.1 ± 17.4	937 ± 351	22.0 ± 13.5	447 ± 239
DM-6722	21.1 ± 10.2	434 ± 214	10.1 ± 4.88	202 ± 96.7

a DLM, delamanid; EMB, ethambutol; *n*, number of subjects; PLC, placebo; Rifater, a combination tablet of rifampin, pyrazinamide, and isoniazid.

**FIG 2:**
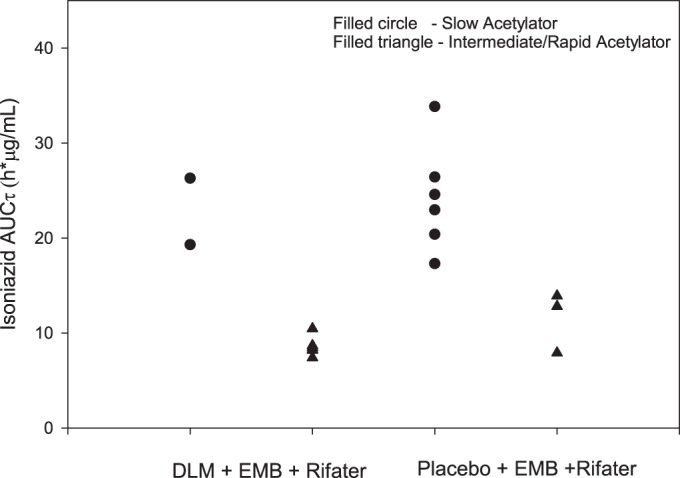
Individual subject steady-state isoniazid AUCτ grouped by acetylator genotype after the administration of ethambutol-Rifater alone or with delamanid. DLM, delamanid; EMB, ethambutol.

With regard to ethambutol concentrations, following coadministration of ethambutol-Rifater with delamanid, equivalence was suggested (Table 3). After the coadministration of ethambutol-Rifater with delamanid, equivalence was suggested for rifampin and pyrazinamide exposures when compared to Rifater given alone (Table 3). With regard to isoniazid, as expected, NAT2 genotype had a profound effect on isoniazid exposure, with slow acetylators having ∼2-fold-higher isoniazid concentrations than intermediate/rapid acetylators. Since the two groups were not matched for genotype, the prospective statistical analysis for isoniazid AUCτ was not interpretable. From visual inspection of individual AUCτ values for slow acetylators compared to intermediate/rapid acetylators, delamanid had no effect on isoniazid PK (Fig. 2).

### (ii) Study 2.

Delamanid concentrations reached steady-state by day 14, the last day of dosing, following 100-mg twice-daily dosing of delamanid alone or with either 300 mg of tenofovir once daily or 400 mg of lopinavir plus 100 mg of ritonavir (Kaletra) twice daily, as indicated by the individual day 12 through day 14 predose delamanid plasma concentrations. Table 2 provides the summary PK data and a statistical evaluation for delamanid. Equivalence in the steady-state exposure of delamanid was documented when coadministered with tenofovir. Equivalence was suggested for delamanid exposure after lopinavir/ritonavir coadministration. When coadministered with delamanid, equivalence was documented for steady-state exposure of lopinavir and suggested for tenofovir and ritonavir (Table 3).

### (iii) Study 3.

Steady-state was reached for delamanid (7 days of 100-mg twice-daily dosing) and efavirenz (10 days of once-daily 600 mg dosing in the evening) concentrations as indicated by the individual predose plasma concentrations. As shown in Table 2 and Fig. 1, efavirenz did not affect the steady-state exposure of delamanid, and delamanid did not affect efavirenz plasma concentrations (Table 3). The efavirenz plasma exposure was in agreement with CYP2B6* genotype (unpublished results).

## DISCUSSION

Delamanid received regulatory approval in the European Union, Japan, and the Republic of Korea for use as part of an appropriate combination drug regimen to treat pulmonary MDR-TB in adults. During the development of delamanid, it was determined that delamanid is primarily metabolized by plasma albumin (15) to form DM-6705 and that delamanid is not metabolized by CYP enzymes (4). Subsequently, the metabolism of DM-6705 is thought to occur via three different pathways, some of which are thought to be mediated by CYP3A4 (24). The *in vitro* data indicated that the lack of involvement of CYP in the primary metabolism of delamanid may be advantageous with regard to potential drug-drug interactions relative to other newer anti-TB agents. However, prediction of *in vivo* interactions from *in vitro* data can be misleading due to complex factors such as heterotropic effects, partial inhibition, nonspecific protein binding, etc., that may bias the extrapolation (25). Therefore, *in vivo* studies to confirm *in vitro* predictions are important, and these three studies were conducted to investigate whether the lack of CYP involvement *in vitro* was reflected in a lack of *in vivo* drug-drug interaction.

**FIG 3:**
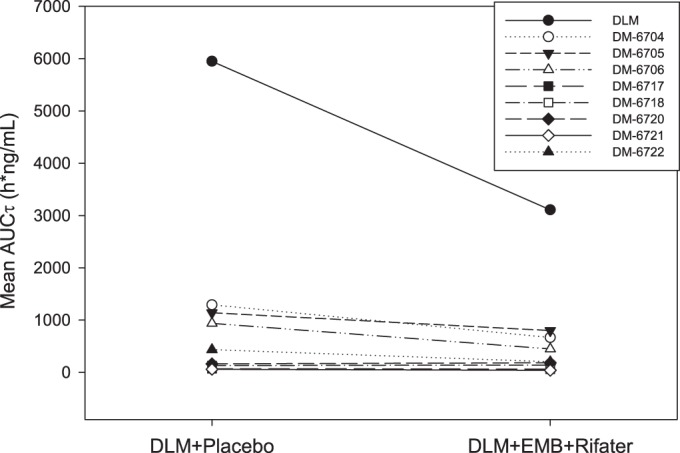
Mean AUCτ (day 15) of delamanid (OPC-67683) and metabolites with or without coadministration of ethambutol-Rifater. DLM, delamanid; DM, delamanid metabolite; EMB, ethambutol.

The anti-TB drug combination of ethambutol-Rifater (isoniazid, rifampin, and pyrazinamide) with delamanid for 15 days in study 1 resulted in about 45% lower exposure of delamanid. Concentrations of the four most prevalent metabolites of delamanid (DM-6704, DM-6705, DM-6706, and DM-6722) were also about 30% and 50% lower relative to delamanid treatment alone (Fig. 3). The ratios of metabolite DM-6704 or metabolite DM-6705 to delamanid were similar between treatments (unpublished results). These observations, coupled with the overall lower concentrations of metabolites, suggest that induction of CYP3A4 by rifampin does not play a major role in the observed lower delamanid exposure with the combination treatment. Rifampin is a potent inducer of cytochrome P450 isozymes, including CYP3A4. If the lower delamanid concentrations with ethambutol-Rifater treatment were due to the induction of CYP3A4, a significant increase in at least one of the metabolites should have been observed. Since the metabolite concentrations were not higher after coadministration with Rifater-ethambutol, the lower delamanid concentrations are likely due to decreased bioavailability of delamanid rather than a change in intrinsic clearance, as would occur after induction by rifampin. In addition, since delamanid coadministered with efavirenz, a CYP3A inducer, did not result in changes in delamanid exposure (study 3), the induction of CYP3A4 is unlikely to have been a major factor for the lower delamanid exposure. Also, considering the low solubility of delamanid, limited absorption of delamanid may have occurred when a total of 15 tablets were ingested by the subjects (including delamanid and the other coadministered medications) within 1.5 h. Induction of MDR1 transporters in the intestine by rifampin is also unlikely since delamanid is neither a substrate of nor an inhibitor of MDR1 (19).

A limitation of study 1 is the fact that a combination of many drugs was given. A more direct evaluation of the potential for interaction with a strong inducer of CYP3A4 would be to study delamanid with a single agent-inducer, such as carbamazepine. However, based on *in vitro* data and the efavirenz data, no significant interaction is expected. Of note, given delamanid's currently approved indication for the treatment of MDR-TB (19), it is not expected to be used with the combination of isoniazid, rifampin, pyrazinamide, and ethambutol, given the current World Health Organization guidelines. However, if such a combination is to be administered with delamanid, caution should be exercised given the decrease in delamanid exposure when administered with the combination. Another limitation is the wide confidence intervals for some of the coadministered drugs, which reflect the relatively limited number of subjects and a parallel-arm design, necessitated by the relatively long half-life of delamanid's metabolites. Study 1 also evaluated the potential effect of delamanid on the coadministered drugs. Delamanid did not meaningfully affect the plasma exposure of coadministered anti-TB drugs isoniazid/rifampin/pyrazinamide or ethambutol.

The antiretroviral drug tenofovir, when coadministered with delamanid, did not affect delamanid or delamanid metabolite systemic exposure in a clinically relevant manner (study 2). Tenofovir is not metabolized by CYP450 isoenzymes, nor is it an inducer or inhibitor of these enzymes. Tenofovir is renally eliminated as unchanged drug by a combination of glomerular filtration and active tubular secretion (26). The small changes seen when coadministered with delamanid are not clinically relevant and are in agreement with the expectation of a lack of interaction based on the metabolic profile of tenofovir and delamanid.

The antiretroviral drug combination of lopinavir/ritonavir (Kaletra), in which ritonavir is a known strong inhibitor of CYP3A4, coadministered with delamanid, resulted in an approximately 25% higher exposure of delamanid and metabolite DM-6705 and an approximately 75% higher exposure of metabolite DM-6704, while other metabolites were lower (study 2, unpublished results). Lopinavir is neither an inhibitor nor an inducer of CYP450 isozymes. Ritonavir is an inhibitor of cytochrome CYP3A and CYP2D6 to a lesser extent. It also appears to induce CYP3A, CYP1A2, CYP2C9, CYP2C19, and CYP2B6, as well as glucuronosyltransferase. Given that ritonavir is a strong inhibitor of CYP3A, if indeed CYP3A were an important pathway for the metabolism of delamanid, there would have been a significant increase in delamanid concentrations. Although there was a small increase in delamanid exposure, it was not clinically meaningful. Further, the inconsistent pattern of metabolite changes, where some of them increased and some decreased, supports the lack of significant CYP3A involvement. Some involvement of CYP3A is possible, as evidenced by the changes in both delamanid concentrations as well as its metabolites.

Efavirenz is metabolized by CYP3A4 and CYP2B6 and is known to inhibit *in vitro* CYP2C9, CYP2C19, and CYP3A4 at therapeutic concentrations. *In vivo*, efavirenz is a moderate inducer of CYP3A4, as well as CYP2B6, and 2C19 (20). In this trial design, as in medical practice, efavirenz was given at night to reduce known AEs. Hence, PK profiles of efavirenz and delamanid were measured after the evening dose and at steady state, namely, after 10 days of efavirenz and after 7 days of delamanid administration. Coadministration of delamanid with efavirenz did not significantly affect delamanid exposure (study 3).

Thus, with regard to the interacting drugs, coadministration of delamanid with tenofovir, lopinavir/ritonavir, or efavirenz did not significantly affect the systemic exposure of these drugs. This is particularly important since combinations of efavirenz, tenofovir, and an additional NRTI are the first line of HIV therapy in many countries where the MDR-TB/HIV coinfection rate is high. The lack of significant CYP-related drug-drug interactions of delamanid in clinical studies is supported by *in vitro* metabolism data. *In vitro* studies showed that metabolites of delamanid were minimally quantifiable after incubation of delamanid with liver microsomes, indicating that delamanid is not metabolized by CYP enzymes and, additionally, that delamanid had neither stimulatory nor inhibitory effects on CYP activities (4). In addition, *in vitro* studies have shown that delamanid is not a substrate of the human transporters MDR1, BCRP, OCT1, OATP1B1, and OATP1B3 and that delamanid does not inhibit the major human transporters MDR1, BCRP, OAT1, OAT3, OCT1, OCT2, OATP1B1, OATP1B3, and BSEP (19).

In consideration of other drugs for MDR-TB that could potentially be administered with delamanid, there are several that are currently being used, such as clofazimine, various fluoroquinolones, ethionamide, prothionamide, cycloserine, paraaminosalicylic acid, polypeptides, aminoglycosides, high-dose isoniazid, and pyrazinamide, which are eliminated by conjugation, acetylation, metabolism by CYP450 isoenzymes, or renal excretion. In addition, other drugs, both new and in development, such as bedaquiline and PA-824, are substrates of and are metabolized by CYP3A (9, 10). Given that delamanid is not a substrate, inhibitor, or inducer of CYP450 isozymes and that it is not conjugated, acetylated, or excreted renally, clinically relevant metabolic interactions with these drugs, if coadministered together, are unlikely.

The drug-drug interaction studies conducted with delamanid show little effect on delamanid exposure due to induction or inhibition of CYP enzymes, which is in agreement with the *in vitro* data. The results of the studies with inducers of CYP3A4 (rifampin and efavirenz) or inhibitors (ritonavir) indicated that CYP3A4 does not play a major role in delamanid's metabolism, a finding in agreement with the *in vitro* data. Also, delamanid has little potential for clinically significant CYP-related drug-drug interactions and may be safely coadministered with other drugs whose metabolism is mediated via the CYP450 pathways.

## References

[1] World Health Organization. 2015 Global tuberculosis report 2015. Document WHO/HTM/TB/2015.22. World Health Organization, Geneva, Switzerland.

[2] U.S. Centers for Disease Control and Prevention. 2014 Reported tuberculosis in the United States, 2013. U.S. Centers for Disease Control and Prevention, Atlanta, GA.

[3] Stop TB Partnership and World Health Organization. 2006 Global plan to stop TB 2006-2015. Document WHO/HTM/STB/2006.35. World Health Organization, Geneva, Switzerland.

[4] Matsumoto M, Hashizume H, Tomishige T, Kawasaki M, Tsubouchi H, Sasaki H, Shimokawa Y, Komatsu M 2006 OPC-67683, a nitro-dihydro-imidazooxazole derivative with promising action against tuberculosis *in vitro* and in mice. PLoS Med 3:e466. doi:10.1371/journal.pmed.0030466. 17132069 PMC1664607

[5] Diacon AH, Dawson R, Hanekom M, Narunsky K, Venter A, Hittel N, Geiter LJ, Wells CD, Paccaly AJ, Donald PR 2011 Early bactericidal activity of delamanid (OPC-67683) in smear-positive pulmonary tuberculosis patients. Int J Tuberc Lung Dis 15:949–954. doi:10.5588/ijtld.10.0616. 21682970

[6] Gler MT, Skripconoka V, Sanchez-Garavito E, Xiao H, Cabrera-Rivero JL, Vargas-Vasquez DE, Gao M, Awad M, Park SK, Shim TS, Suh GY, Danilovits M, Ogata H, Kurve A, Chang J, Suzuki K, Tupasi T, Koh WJ, Seaworth B, Geiter LJ, Wells CD 2012 Delamanid for multidrug-resistant pulmonary tuberculosis. N Engl J Med 366:2151–2160. doi:10.1056/NEJMoa1112433. 22670901

[7] Skripconoka V, Danilovits M, Pehme L, Tomson T, Skenders G, Kummik T, Cirule A, Leimane V, Kurve A, Levina K, Geiter LJ, Manissero D, Wells CD 2013 Delamanid improves outcomes and reduces mortality in multidrug-resistant tuberculosis. Eur Respir J 41:1393–1400. doi:10.1183/09031936.00125812. 23018916 PMC3669462

[8] Gupta R, Geiter LJ, Wells CD, Gao M, Cirule A, Xiao H 2015 Delamanid for extensively drug-resistant tuberculosis. N Engl J Med 373:291–292. doi:10.1056/NEJMc1415332. 26176402

[9] van Heeswijk RPG, Dannemann B, Hoetelmans RM 2014 Bedaquiline: a review of human pharmacokinetics and drug-drug interactions. J Antimicrob Chemother 69:2310–2318. doi:10.1093/jac/dku171. 24860154

[10] Dooley KE, Luetkemeyer AF, Park JG, Allen R, Cramer Y, Murray S, Sutherland D, Aweeka F, Koletar SL, Marzan F, Bao J, Savic R, Haas DW, AIDS Clinical Trials Group A5306 Study Team. 2014 Phase I safety, pharmacokinetics, and pharmacogenetics study of the antituberculosis drug PA-824 with concomitant lopinavir-ritonavir, efavirenz, or rifampin. Antimicrob Agents Chemother 58:5245–5252. doi:10.1128/AAC.03332-14. 24957823 PMC4135849

[11] Niemi M, Backman JT, Fromm MF, Neuvonen PJ, Kivistö KT 2003 Pharmacokinetic interactions with rifampicin: clinical relevance. Clin Pharmacokinet 42:819–850. doi:10.2165/00003088-200342090-00003. 12882588

[12] Usach I, Melis V, Peris JE 2013 Non-nucleoside reverse transcriptase inhibitors: a review on pharmacokinetics, pharmacodynamics, safety and tolerability. J Int AIDS 16:1–14. doi:10.7448/IAS.16.1.18567. PMC3764307 24008177

[13] Hsu A, Granneman GR, Bertz RJ 1998 Ritonavir. Clinical pharmacokinetics and interactions with other anti-HIV agents. Clin Pharmacokinet 35:275–291. doi:10.2165/00003088-199835040-00002. 9812178

[14] Relling MV 1989 Polymorphic drug metabolism. Clin Pharmacy 8:852–863. 2689060

[15] Shimokawa Y, Sasahara K, Koyama N, Kitano K, Shibata M, Yoda N, Umehara K 2015 Metabolic mechanism of delamanid, a new anti-tuberculosis drug, in human plasma. Drug Metab Dispos 43:1277–1283. doi:10.1124/dmd.115.064550. 26055621

[16] U.S. Food and Drug Administration, HHS. 9 5 1997 International Conference on Harmonisation: Good clinical practice: consolidated guideline; notice of availability. Fed Regist 62:25692–25709. https://www.gpo.gov/fdsys/pkg/FR-1997-05-09/pdf/97-12138.pdf.

[17] Stat-Trade, Inc. 2007 Myambutol (ethambutol hydrochloride), United States, product insert. Stat-Trade, Inc, Northport, NY http://www.accessdata.fda.gov/drugsatfda_docs/label/2008/016320s063lbl.pdf.

[18] Sanofi-Aventis. 2013 Rifater (rifampin, isoniazid, pyrazinamide), United States, product insert. Sanofi-Aventis, Bridgewater, NJ http://products.sanofi.us/rifater/Rifater.pdf.

[19] Otsuka Novel Products GmbH. 2014 Deltyba (delamanid) annex I: summary of product characteristics. Otsuka Novel Products GmbH, Munich, Germany http://www.ema.europa.eu/docs/en_GB/document_library/EPAR_-_Product_Information/human/002552/WC500166232.pdf.

[20] Bristol-Myers Squibb Company. 2015 Sustiva (efavirenz), United States, product Insert. Bristol-Myers Squibb Company, Princeton, NJ http://packageinserts.bms.com/pi/pi_sustiva.pdf.

[21] Meng M, Smith B, Johnston B, Carter S, Brisson J, Roth SE 2015 Simultaneous quantitation of delamanid (OPC-67683) and its eight metabolites in human plasma using UHPLC-MS/MS. J Chromatogr B 1002:78–91. doi:10.1016/j.jchromb.2015.07.058. 26319300

[22] Jusko WJ 1992 Guidelines for collection and analysis of pharmacokinetic data, p 1–43. *In* Evans WE, Schentag JJ, Jusko WJ (ed), Applied pharmacokinetics: principles of therapeutic drug monitoring, 3rd ed Applied Therapeutics, Vancouver, WA.

[23] Williams RL, Chen ML, Hauck WW 2002 Equivalence approaches. Clin Pharmacol Ther 72:229–37. doi:10.1067/mcp.2002.126705. 12235443

[24] Sasahara K, Shimokawa Y, Hirao Y, Koyama N, Kitano K, Shibata M, Umehara K 2015 Pharmacokinetics and metabolism of delamanid, a novel anti-tuberculosis drug, in animals and humans: importance of albumin metabolism *in vivo*. Drug Metab Dispos 43:1267–1276. doi:10.1124/dmd.115.064527. 26055620

[25] Wienker LC, Heath TG 2005 Predicting *in vivo* drug interactions from *in vitro* drug discovery data. Nat Rev Drug Discov 4:825–833. doi:10.1038/nrd1851. 16224454

[26] Kearney BP, Flaherty JF, Shah J 2004 Tenofovir disoproxil fumarate: clinical pharmacology and pharmacokinetics. Clin Pharmacokinet 43:595–612. doi:10.2165/00003088-200443090-00003. 15217303

